# Impact of thoracic tumor radiotherapy on survival in non‐small‐cell lung cancer with malignant pleural effusion treated with targeted therapy: Propensity score matching study

**DOI:** 10.1002/cam4.6130

**Published:** 2023-06-08

**Authors:** Qingsong Li, Cheng Hu, Shengfa Su, Zhu Ma, Yichao Geng, Yinxiang Hu, Haijie Jin, Huiqin Li, Bing Lu

**Affiliations:** ^1^ Department of Thoracic Oncology Affiliated Hospital of Guizhou Medical University Guiyang China; ^2^ Department of Thoracic Oncology Affiliated Cancer Hospital of Guizhou Medical University Guiyang China; ^3^ Teaching and Research Department of Oncology Clinical Medical College of Guizhou Medical University Guiyang China

**Keywords:** ALK‐positive (ALK‐P), EGFR‐mutant (EGFR‐M), malignant pleural effusion (MPE), non‐small‐cell lung cancer (NSCLC), overall survival (OS), thoracic tumor radiotherapy

## Abstract

**Background:**

EGFR‐mutant (EGFR‐M) and ALK‐positive (ALK‐P)are common in malignant pleural effusion (MPE) with metastatic non‐small‐cell lung cancer (NSCLC) (MPE‐NSCLC). The impact of thoracic tumor radiotherapy on survival in such patients remains unclear. We aimed to investigate whether thoracic tumor radiotherapy could improve overall survival (OS) in such patients.

**Methods:**

According to whether or not patients accepted thoracic tumor radiotherapy, 148 patients with EGFR‐M or ALK‐P MPE‐NSCLC treated with targeted therapy were classified into two groups: DT group without thoracic tumor radiotherapy and DRT group with thoracic tumor radiotherapy. Propensity score matching (PSM) was performed to balance clinical baseline characteristics. Overall survival was analyzed by Kaplan–Meier, compared by log‐rank test, and evaluated using Cox proportional hazards model.

**Results:**

Median survival time (MST) was 25 months versus 17 months in the DRT group and DT group. The OS rates at 1, 2, 3, 5 years in the DRT group and DT group were 75.0%, 52.8%, 26.8%, 11.1% and 64.5%, 28.4%, 9.2%, 1.8%, respectively (χ^2^ = 12.028, *p* = 0.001). Compared with DT group, the DRT group still had better survival after PSM (*p* = 0.007). Before and after PSM, factors associated with better OS through multivariable analysis were that thoracic tumor radiotherapy, radiotherapy, N_0‐2_, and ALK‐TKIs. Grades 4–5 radiation toxicities were not observed in patients; 8 (11.6%) and 7 (10.1%) out of the DRT group suffered from Grade 3 radiation esophagitis and radiation pneumonitis, respectively.

**Conclusion:**

Our results for EGFR‐M or ALK‐P MPE‐NSCLC showed that thoracic tumor radiotherapy may be crucial factor in improving OS with acceptable toxicities. Potential biases should not be neglected: Further randomized controlled trials are necessary to confirm this result.

## 1 INTRODUCTION

Malignant pleural effusion (MPE) is common in patients with advanced stage of all kinds of caners, the most common of which is lung cancer.^1^ Non‐small‐cell lung cancer (NSCLC, rate 86%)^2^ is the dominant pathological type. Malignant pleural effusion is related to a worse prognosis^3^, ^4^ and a decrease in quality of life.^5^, ^6^ According to the guidelines of the National Comprehensive Cancer Network (NCCN) and other authoritative guidelines,^7^ treatment for NSCLC with MPE (MPE‐NSCLC) mainly include two goals: The first goal is to control all involving lesions with systemic treatment and the second is to improve quality of life through the local treatment of MPE.

Systemic treatment for stage IV NSCLC has entered the era of precision therapy, including chemotherapy,^8^ targeted therapy,^9^ and immunotherapy.^10^, ^11^ Tyrosine kinase inhibitors (TKIs) can achieve an excellent therapeutic response in NSCLC with positive mutations in driver Genes and significantly improve progression‐free survival (PFS).^12^, ^13^, ^14^, ^15^ However, disease progression is an inevitable fatal flaw of targeted therapy and occurs mainly within 2 years.^16^, ^17^ Suchit H. Patel et al. and Al‐Halabi H et al. found that the predominant failure in EGFR‐mutant (EGFR‐M)NSCLC without MPE is in the initial involved lesions (Rate, 79.6–82.4%).^18^, ^19^ Multiple studies^20^, ^21^, ^22^, ^23^, ^24^, ^25^ have demonstrated that local therapies, such as radiotherapy, can significantly increase progression‐free survival (PFS) and overall survival (OS).

However, all of the above studies excluded patients with MPE‐NSCLC. We aimed to investigate whether thoracic tumor radiotherapy could improve OS in such patients and to provide evidence for further prospective clinical trials.

## 2 MATERIALS AND METHODS

### 2.1 Patients

A retrospective study was conducted on patients with EGFR‐M or anaplastic lymphoma kinase‐positive (ALK‐P) NSCLC who had MPE from January 2010 to December 2020 at Affiliated Cancer Hospital of Guizhou Medical University (American Joint Committee on Cancer staging system 7th edition). We searched for potential patients in the electronic medical records system by several keywords: targeted drugs available in our hospital, including gefitinib, icotinib, erlotinib, osimertinib, crizotinib, and alectinib. The potential patients who met the following criteria were enrolled: (1) histologically or cytologically confirmed MPE‐NSCLC and molecular testing confirmed EGFR‐M or ALK‐P; (2) treated with targeted therapy; (3) age ≥18 years old; (4) without history of thoracic tumor radiotherapy or/and thoracic surgery; (5) without history of malignancy or/and other concomitant malignancy; (6) MPE controlled by the medical intervention (systemic treatment, *n* = 41; systemic treatment plus Intrathoracic chemotherapy, *n* = 107).

Through the electronic medical records and the department of epidemiology, we acquired patients characteristics including age, sex, Karnofsky performance status (KPS), pathological type, TNM status, positive mutations, metastasis status, and subsequent treatment. The data of positive mutations were inputted according to electronic medical records of our hospital. This retrospective study was approved by the ethics committee of the affiliated cancer Hospital of Guizhou medical university.

Depending on whether or not patients accepted thoracic tumor radiotherapy, patients were classified into two groups: the DT group without thoracic tumor radiotherapy (primary tumor+lymph nodes) and the DRT group with thoracic tumor radiotherapy.

### 2.2 Radiotherapy

#### 2.2.1 Thoracic tumor radiotherapy

Thoracic tumor radiotherapy was not coercive for MPE‐NSCLC, but it was performed when MPE was under control and the patient had signed an informed consent form. The treating radiotherapist made the radiotherapy regime. The prescribed dose was to be at least 30 Gy and given according to the tolerance of the organ at risk. Seventy‐two patients received external beam radiation therapy (EBRT) for thoracic tumor (30Gy ≤ dose < 45Gy, *n* = 6; 45Gy ≤ dose <54 Gy, *n* = 16; 54Gy ≤ dose ≤ 63Gy, *n* = 50). Radiotherapy toxicities were assessed and graded in accordance with Common Terminology Criteria for Adverse Events (CTCAE 4.0).

#### 2.2.2 Metastases radiotherapy

Metastatic radiotherapy depended on clinical need, such as brain metastasis, bone metastasis with moderate–severe pain or risk of fracture, and vertebral metastasis with risk of compressive myelopathy . Brain metastases radiotherapy included whole brain radiotherapy (WBRT) (30Gy/10 fractions or 40Gy/20 fractions), simultaneous integrated boost intensity‐modulated radiotherapy (SIB‐IMRT) (Boost, 47‐48Gy/10–12 fractions, WBRT 30Gy/10–12 fractions), and fractionated stereotactic radiotherapy (FSRT) (50Gy/10fractions). Bone metastases radiotherapy was given in 3Gy per fraction (once a day, 5 days a week, total dose 30–45 Gy).

### 2.3 Statistical analysis

The study employed SPSS software (version 26.0) for statistical analysis. Pearson's chi‐squared or Fisher's exact test was used to assess baseline characteristics. Overall survival^26^ was defined as the period from start date of treatment (radiotherapy execution date for 31 patients with rescue radiation and start date of any treatment for the remaining patients) to the last follow‐up date or death from any cause. Propensity score matching (PSM, ratio 1:1, match tolerance = 0.1) was performed to minimize the impact of potential confounding factors. The PSM was calculated using logistic regression based on 14 pretherapy factors (age, sex, histology, smoking status, KPS, T stage, N stage, metastasis status [MPE only vs. MPE plus other metastases], involving organ (≤3 vs. >3) bone metastases, brain metastases, lung metastases, liver metastases, and the positive mutation types [EGFR vs. ALK]), and one therapy factor (systematic chemotherapy). These factors may affect survival. Overall survival was analyzed by Kaplan–Meier, compared by log‐rank test, and evaluated using Cox proportional hazards model. All statistical tests were two‐sided, and *p*‐values <0.05 were considered statistically significant.

## 3 RESULTS

### 3.1 Patient characteristics

**FIGURE 1:**
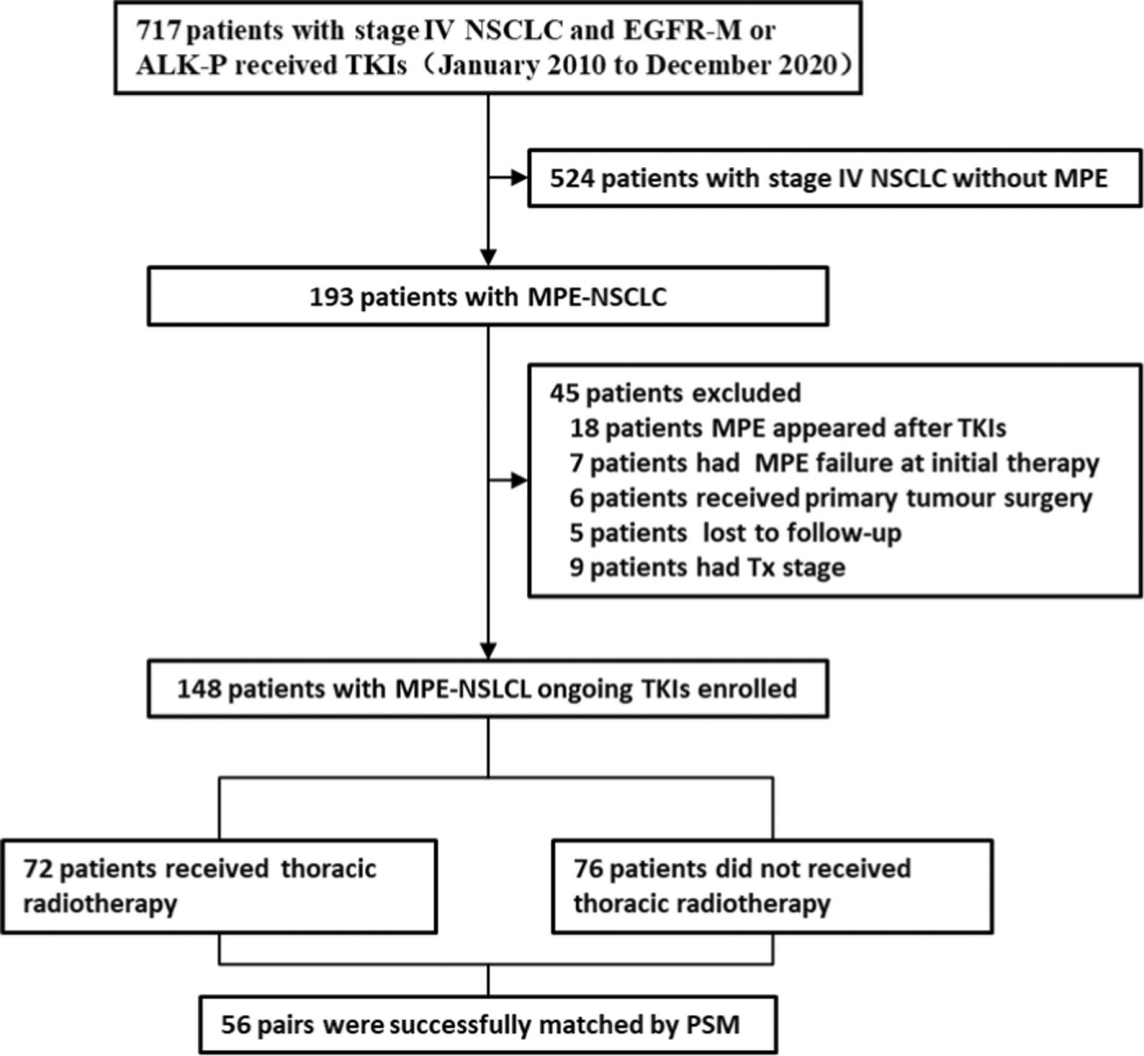
Flowchart of patient cohort.

From January 2010 to December 2020, a total of 148 patients were statistically analyzed. Of 148 patients, 72 accepted thoracic tumor radiotherapy (DRT group, Figure 1). Clinical characteristics are presented in Table 1. Males accounted for 48.6% of the total; the median age at MPE diagnosis was 56 years (age ≤70 years in 124 patients), and the KPS of about two‐thirds of patients was less than or equal to 80. T stage distribution at diagnosis was T_1–2_(*n* = 46, 31%) and T_3–4_(*n* = 102, 69%), the corresponding N stage was N_0–2_(*n* = 65, 44%) and (*n* = 83, 56%). At initial diagnosis, the top three metastatic organs were bone (43.9%), lung (28.4%), and brain (21.6%). The two groups were no significant difference concerning sex, age, smoking status, KPS, T stage, N stage, metastasis status (MPE only vs MPE plus other metastases), involving organ (≤3 vs. >3), and positive mutation types (EGFR‐M vs. ALK‐P). Most patients (*n* = 107, 72.3%) underwent one‐ to four‐cycle intrathoracic chemotherapy (median: 1 cycle) in the initial treatment. Sixty‐four patients underwent systematic chemotherapy (*n* = 47, initial treatment; *n* = 15, disease progression). Disease progression was observed in 59 patients in DT group and 26 patients undergone local failure Table S1.

**TABLE 1:** Clinical characteristics in 148 patients.

		All	DRT group	DT group	χ^2^	*p* value
		(*n* = 148)	(*n* = 72)	(*n* = 76)		
Sex	Male	72	35	37	0.000	>0.999
	Female	76	37	39		
Age (years)	≤56	76	42	34	2.736	0.104
	>56	72	30	42		
Histology (squamous carcinoma)	Yes	2	2	0	—	0.235
	No	146	70	76		
Smoking	Yes	54	27	27	0.062	0.865
	No	94	45	49		
KPS	≤80	95	46	49	0.06	>0.999
	90–100	53	26	27		
T stage	≤2	46	22	24	0.018	>0.999
	3–4	102	50	52		
N stage	≤2	65	34	31	0.621	0.508
	3	83	38	45		
Metastatic status	MPE only	41	23	18	1.260	0.276
	MPE + other metastasis	107	49	58		
Other metastases						
Bone	Yes	65	28	37	1.440	0.250
	No	83	44	39		
Brain	Yes	32	15	17	0.051	0.844
	No	116	57	59		
Lung	Yes	42	16	26	2.614	0.144
	No	106	56	50		
Liver	Yes	13	3	10	3.731	0.080
	No	135	69	66		
Adrenal	Yes	10	5	5	—	>0.999
	No	138	67	71		
Other	Yes	25	13	12	0.135	0.827
	No	123	59	64		
Involving organ≤3	Yes	53	27	26	0.174	0.733
	No	95	45	50		
Positive mutation types	EGFR‐M	127	62	65	0.010	>0.999
	ALK‐P	21	10	11		
Systematic chemotherapy	Yes	64	42	22	13.009	<0.001
	No	84	30	54		
EGFR‐TKIs	Gefitinib^1^/Icotinib^2^/Erlotinib^3^	110	53	57	0.133	0.797
	Osimertinib after 1/2/3	17	9	8		
ALK‐TKIs	Crizotinib	17	8	9	—	>0.999
	Alectinib/Alectinib after Crizotinib	4	2	2		
Radiotherapy						
Thoracic tumor^b^	EBRT (30–63 Gy)	72				
Metastases^a^		56				
Brain		33				
	FSRT (50Gy/10f)	18				
	Whole‐brain irradiation	8				
	SIB‐IMRT	7				
Bone	EBRT	14				
Other metastases	EBRT	9				

Abbreviations: FSRT, fractionated stereotatic radiotherapy; KPS, Karnofsky performance status; SIB‐IMRT, Simultaneous Integrated Boost intensity modulated radiation therapy.

^a^ Nine patients treated with other site radiotherapy (bone in 6 patients, other metastasis in 3 patients).

^b^ Thoracic tumor, primary tumor+lymph nodes.

### 3.2 Radiotherapy

Of the 148 patients, 29.7% (44 patients) underwent radiotherapy at both thoracic tumor radiotherapy and metastasis sites, 27.0% (40 patients) at either thoracic tumor or metastasis sites, but 43.3% (64 patients) did not receive any radiotherapy (Table 1). Thoracic tumor radiotherapy and targeted therapy were performed concurrently in 41 patients (concurrent chemotherapy in 31 patients). Of the 72 patients in the DRT group, 73.6% (*n* = 53) received thoracic tumor radiotherapy in the initial treatment and 26.4% (*n* = 19) received rescue radiation after disease progression. In the DT group, 15.8% (*n* = 12) received rescue radiation after disease progression.

### 3.3 Survival outcomes

**FIGURE 2:**
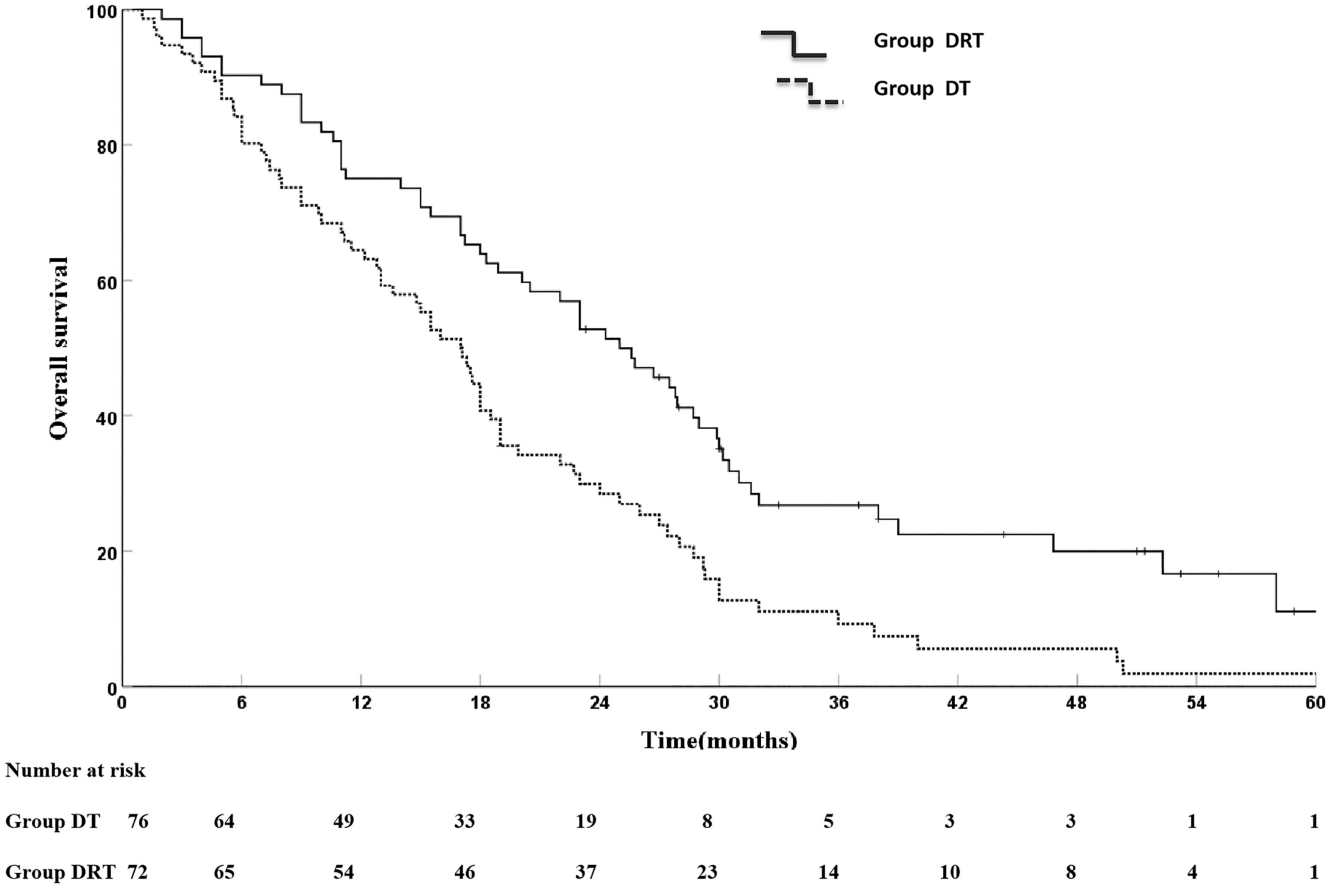
Overall survival outcomes in DRT and DT groups.

The last follow‐up was in November 2022, with median follow‐up of 22 months. At last follow‐up, 126 of 148 cases died. Median survival time (MST)of 148 patients was 18.9 months (95% CI: 15.6–22.2), and 1 year, 2 years, 3 years, and 5 years of OS rates were 69.6%, 10.3%, 18.0%, and 6.7%. The OS rates at 1, 2, 3, 5 years of the DRT group and DT group were 75.0%, 52.8%, 26.8%, 11.1% and 64.5%, 28.4%, 9.2%, 1.8%, respectively; corresponding MST was 25 months in the DRT group and 17 months in the DT group (χ^2^ = 12.028, *p* = 0.001) (Figure 2). Radiotherapy at any sites significantly improved OS (*n* = 84, MST 22.0 vs. 17.5 months, χ^2^ = 4.904, *p* = 0.027).

### 3.4 OS analysis after PSM

**FIGURE 3:**
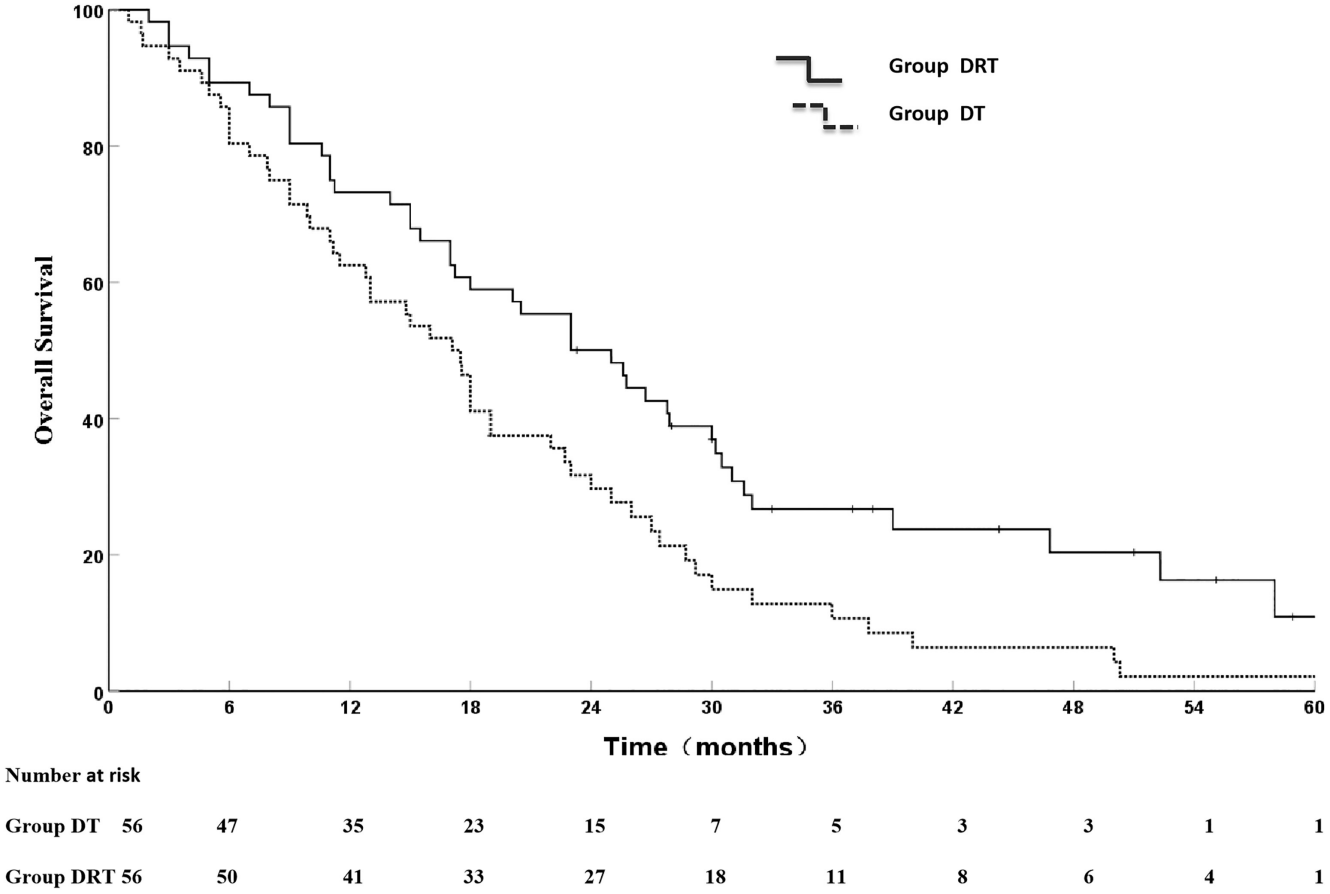
Overall survival outcomes in DRT and DT groups after PSM.

Following the PSM, 56 pairs were successfully matched. After PSM, the baseline characteristics were shown in Table S2. The survival outcomes were in keeping with that before PSM. The OS rates at 1, 2, 3, 5 years of the DRT group and DT group were 73.2%, 50.0%, 26.7%, 10.8% and 62.5%, 29.7%, 10.7%, 2.1%, respectively. The MST was 23 months and 17.1 months in the DRT group and DT group (χ^2^ = 7.250, *p* = 0.007) (Figure 3).

### 3.5 Univariate and multivariate analysis

Univariate analysis showed that T_1‐2_ stage, N_0‐2_ stage, MPE only, No brain metastases, No liver metastases, Involving organ ≤3, ALK‐TKIs, radiotherapy, and thoracic tumor radiotherapy were significantly related with more excellent OS (Table 2). Multivariate analysis revealed that thoracic tumor radiotherapy, radiotherapy, T_1‐2_, N_0‐2_, and ALK‐TKIs were independent predictors of better OS. In multivariate analysis after PSM, thoracic tumor radiotherapy, radiotherapy, N_0‐2_, and ALK‐ TKIs predicted longer OS (Table 3).

**TABLE 2:** Univariable analysis on OS before and after PSM.

Variable		Before PSM (=148)			After PSM (*n* = 112)		
		HR	95% CI	p	HR	95% CI	*p*
Sex	Male vs. Female	0.95	0.67–1.34	0.758	1.06	0.71—1.59	0.786
Age	≤56 vs. >56	0.86	0.60–1.22	0.384	0.87	0.58–1.31	0.511
Smoking	Yes vs. No	1.15	0.80–1.65	0.450	1.19	0.79–1.80	0.396
KPS	≤80 vs. >80	1.33	0.92–1.92	0.130	1.21	0.80–1.83	0.357
T status	T_1–2_ vs. T_3–4_	0.58	0.39–0.87	0.008	0.63	0.40–0.98	0.039
N status	N_0–2_ vs. N_3_	0.67	0.47–0.97	0.032	0.59	0.39–0.89	0.012
MPE only	Yes vs. No	0.59	0.40–0.89	0.012	0.62	0.39–0.96	0.033
Bone metastases	Yes vs. No	1.33	0.93–1.89	0.117	1.26	0.84–1.89	0.267
Brain metastases	Yes vs. No	1.54	1.00–2.35	0.046	1.42	0.87–2.34	0.164
Lung metastases	Yes vs. No	1.32	0.90–1.95	0.157	1.21	0.78–1.88	0.391
Liver metastases	Yes vs. No	1.98	1.09–3.62	0.026	2.02	0.88–4.64	0.099
Adrenal metastases	Yes vs. No	0.94	0.48–1.86	0.867	0.98	0.45–2.13	0.963
Other metastases	Yes vs. No	0.90	0.56–1.46	0.678	1.0.06	0.63–1.80	0.819
Involving organ	≤3 vs. >3	0.67	0.46–0.97	0.014	0.64	0.42–0.97	0.037
TKIs	EGFR vs. ALK	2.22	1.24–3.95	0.007	1.79	0.93–3.45	0.084
Systematic chemotherapy	Yes vs. No	0.81	0.57–1.15	0.238	1.02	0.69–1.54	0.902
thoracic radiotherapy	Yes vs. No	0.54	0.38–0.77	0.001	0.58	0.38–0.87	0.008
Radiotherapy	Yes vs. No	0.67	0.47–0.96	0.029	0.73	0.49–1.09	0.123
Metastases radiotherapy	Yes vs. No	0.85	0.59–1.23	0.392	0.81	0.53–1.24	0.332

Abbreviations: CI, confidence interval; HR, hazard ratio; PSM, propensity score matching.

**TABLE 3:** Multivariate analysis of overall survival.

Variable	Before PSM			After PSM		
	HR	95% CI	*p*	HR	95% CI	*p*
Thoracic tumor radiotherapy (yes vs. no)	0.214	0.104–0.440	<0.001	0.252	0.115–0.553	0.001
T1‐2 vs. T3‐4	0.615	0.412–0.920	0.018			
N 0–2 vs. N3	0.688	0.475–0.996	0.047	0.548	0.356–0.845	0.007
ALK‐TKIs vs. EGFR‐TKIs	0.336	0.180–0.627	0.001	0.430	0.212–0.871	0.019
Radiotherapy (yes vs. no)	0.333	0.163–0.683	0.003	−0.383	0.172–0.853	0.019

### 3.6 Lung radiation toxicity

The majority of lung radiation toxicities were Grade 1. From our observation, patients did not exhibit Grades 4–5 radiation toxicities. In 69 evaluable patients, the rates of Grade 3 radiation pneumonitis (RP) and esophagitis (RE) were 10.1% and 11.6%, respectively. There were 12 (17.4%) and 15 (21.7%) cases of Grade 2 RP and RE, respectively.

## 4 DISCUSSION

For all we know, this is the first research to report the role of thoracic tumor radiotherapy on OS in MPE‐NSCLC patients treated with targeted therapy. The clinical characteristics of these patients included male proportion of 48.6%, age ≤70 years in majority of the patients, distant metastasis site, and T_3–4_ and N_3_ for 68.9% and 56.1% of patients, respectively, which were analogous to those reported for stage IV NSCLC without MPE.^27^, ^28^, ^29^ Treatment for MPE‐NSCLC mainly aims at controlling MPE to improve life quality based on systematic drug treatment. However, the prognosis was poor, with the mean survival time (MST) of 4 months for systematic chemotherapy^3^, ^4^ and 16–18 months for simple EGFR‐TKIs.^27^, ^30^, ^31^

Historically, platinum‐based chemotherapy was the first‐line standard treatment for advanced NSCLC. Higginson DS et al. found that for such patients accepting systemic chemotherapy alone, large central tumor, bronchial compression, and vascular compression were related to poor OS.^32^ The predominant cause of death in advanced NSCLC is disease progression of thoracic tumor.^33^ During the past decade, several studies have demonstrated that for stage IV NSCLC without MPE, thoracic tumor radiotherapy combined with systematic drug treatment can decrease the local progression rate to 30% and significantly extend OS.^24^, ^34^, ^35^, ^36^, ^37^ Similar to outcome of platinum‐based chemotherapy, several clinical trials have demonstrated that the local failure rate in stage IV NSCLC with EGFR‐M or ALK‐P treated with targeted therapy alone is greater than 80%.^18^, ^19^ In the era of targeted therapy, several studies have proved that thoracic tumor radiotherapy improves OS^25^, ^38^, ^39^ for patients with NSCLC without MPE. However, there is no evidence that thoracic tumor radiotherapy improves OS for MPE‐NSCLC treated with targeted therapy.

EGFR‐TKIs has become an vital systemic treatment in patients with MPE‐NSCLC on account of adenocarcinoma as the predominant pathological type,^28^ which has a high rate of EGFR‐M, especially in the Asian population.^40^ Yang J et al.^41^ found that the ORRs for target lesions and MPE were 88.5% and 61.5%, respectively. Previous studies^27^, ^30^, ^31^ showed that for MPE‐NSCLC patients treated with targeted therapy, the MST was approximately 16 months, in accord with survival outcomes in our study (17 months). Our study showed that the DRT group had significantly better OS in comparison with the D group (MST, 25 months versus 17 months, *p* = 0.001). However, our study had some unaccounted confounders, selection bias, and recall bias, which are the inevitable nature of retrospective research. PSM can reduce confounders and bias, and might make our results more reliable. Figure 3 depicted that the OS outcome following PSM was similar to that before PSM (56 pairs, *p* = 0.007). The OS of MPE‐NSCLC patients is short, and one of the most critical adverse factors for OS is a large lung mass.^42^ The more superior survival of the DRT group might be attributed to thoracic tumor controlled continuously, reduction in thoracic tumor failure^39^, ^43^ and additional disease control after progression.^20^, ^44^ As reported in previous studies, the OS outcome was in keeping with stage IV NSCLC without MPE based on multimodal treatment, including thoracic tumor radiotherapy and targeted therapy.^25^, ^38^, ^39^

Multivariate analysis revealed that thoracic tumor radiotherapy was independently associated with the extend of OS in such patients. Our findings recommended that the treatment pattern of EGFR‐M/ALK‐P MPE‐NSCLC should not be restricted to a monotherapy but rather be a multimodal treatment. Overall survival may benefit from the synergistic combination^45^, ^46^, ^47^ of local radiotherapy and targeted therapy. Another result of the multivariate analysis was that N_0‐2_ was closely relevant to improving OS in such patients. It indicated that early lymph node staging was associated with a good prognosis.^48^ The study by Parikh RB et al.^49^ also found that N_2‐3_ was associated with a greater hazard of death. A retrospective study^50^ indicated that fewer metastatic lymph nodes were associated with better OS for NSCLC.

It is acknowledged that the synergistic combination of local radiotherapy and targeted therapy for NSCLC lead to increased toxicity in comparison with monotherapy. Radiation toxicities of Grade 3 were observed in approximately 10% of patients, analogous to that of synchronous/concurrent chemoradiotherapy^36^, ^48^, ^51^ or EGFR‐TKIs with radiotherapy^52^, ^53^ in stage IV NSCLC without MPE. This reveals that radiation toxicities are acceptable in such patients, but it is essential to minimize the incidence and severity of RP as much as possible.^47^, ^54^

Although the results of our study have very significant clinical implications, there are several shortcomings. First of all, the samples were all inpatients because of outpatients are not searchable through electronic medical records, which may lead to selection bias. Second of all, although this was a population study of hospitalized patients in our hospital, single institution is a disadvantage. Third of all, systematic treatment in the first line included targeted therapy (*n* = 101, 68.2%) and systematic chemotherapy (*n* = 47, 31.8%). Finally, this was a retrospective study with inevitable shortcomings despite using a PSM method. Therefore, randomized controlled trials (RCT) are necessary to validate the findings of this study.

## 5 CONCLUSIONS

To date, the treatment of MPE‐NSCLC has not changed over the past decades. Local treatment has always been a taboo area in MPE‐NSCLC. Our results for EGFR‐M or ALK‐P MPE‐NSCLC showed that thoracic tumor radiotherapy may be crucial factor in improving OS with acceptable toxicities. Potential biases should not be neglected: Further randomized controlled trials are necessary to confirm this result. Exploration of local treatment must be based on guidelines, with the aim of prolonging survival while maintaining quality of life.

## AUTHOR CONTRIBUTIONS

**Qingsong Li:** Conceptualization (equal); data curation (equal); formal analysis (equal); investigation (equal); methodology (equal); resources (equal); software (equal); validation (equal); visualization (equal); writing – original draft (equal); writing – review and editing (equal). **Cheng Hu:** Data curation (equal); formal analysis (equal); investigation (equal); resources (equal); validation (equal); visualization (equal); writing – original draft (equal). **Sheng‐Fa Su:** Conceptualization (equal); formal analysis (equal); project administration (equal); supervision (equal); writing – review and editing (equal). **Zhu Ma:** Investigation (equal); methodology (equal); resources (equal). **Yichao Geng:** Formal analysis (equal); investigation (equal); methodology (equal); resources (equal); validation (equal). **Yinxiang Hu:** Data curation (equal); investigation (equal); resources (equal); supervision (equal); validation (equal). **Haijie Jin:** Data curation (equal); investigation (equal); resources (equal); writing – original draft (equal). **Huiqin Li:** Investigation (equal); resources (equal). **Bing Lu:** Conceptualization (equal); data curation (equal); formal analysis (equal); funding acquisition (equal); investigation (equal); methodology (equal); project administration (equal); resources (equal); software (equal); supervision (equal); validation (equal); visualization (equal); writing – review and editing (equal).

## FUNDING INFORMATION

Guizhou Science and Technology Plan Support Project [Qiankehe support (2019) 2795].

## CONFLICT OF INTEREST STATEMENT

None.

## ETHICS STATEMENT

This study was reviewed by the ethical review boards in China (Ethics Committee of Affiliated Cancer Hospital of Guizhou Medical University, GuiYang, China).

## Supporting information


Table S1.

Table S2.

## Data Availability

The raw data supporting the conclusions of this article will be made available by the authors, without undue reservation.
