# Supplementary material for: Impact of thoracic tumor radiotherapy on survival in non‐small‐cell lung cancer with malignant pleural effusion treated with targeted therapy: Propensity score matching study
Source: Cancer Med. 2023 Jun 8;12(14):14949–59. doi: 10.1002/cam4.6130 (PMC10417183; doi:10.1002/cam4.6130)
Supplement: Supplementary file 1 — Table S1. [file CAM4-12-14949-s001.docx]

Table1 Disease progression in 148 patients

|  |  | All | DRT group | DT group |
| --- | --- | --- | --- | --- |
|  |  | n=148 | n=72 | n=76 |
| Disease progression | Unknown | 22 | 8 | 14 |
|  | No | 18 | 15 | 3 |
|  | Yes | 108 | 49 | 59 |
| Radiotherapy after disease progression | thoracic tumour* | 10 | 10 | 0 |
|  | distant sites | 12 | 0 | 12 |
|  | Both sites | 9 | 9 | 0 |
| Radiotherapy at both initial treatment and after disease progression | thoracic tumour r | 1 | 1 | 0 |
|  | distant sites | 12 | 12 | 0 |

*Thoracic tumour ,primary tumor+lymph nodes
