# Supplementary material for: Impact of thoracic tumor radiotherapy on survival in non‐small‐cell lung cancer with malignant pleural effusion treated with targeted therapy: Propensity score matching study
Source: Cancer Med. 2023 Jun 8;12(14):14949–59. doi: 10.1002/cam4.6130 (PMC10417183; doi:10.1002/cam4.6130)
Supplement: Supplementary file 2 — Table S2. [file CAM4-12-14949-s002.docx]

Patients characteristics after PSM

| characteristic | | All | DRT group | DT group | χ*^2^* | *P* value |
| --- | --- | --- | --- | --- | --- | --- |
|  |  | (n=112) | (n=56) | (n=56) |  |  |
| Sex | Male | 60 | 29 | 31 | 0.144 | 0.850 |
|  | Female | 52 | 27 | 25 |  |  |
| Age (years) | ≤56 | 60 | 31 | 29 | 0.144 | 0.850 |
|  | ＞56 | 52 | 25 | 27 |  |  |
| Histology | non squamous carcinoma | 108 | 56 | 56 | - | - |
| Smoking | Yes | 44 | 21 | 23 | 0.150 | 0.847 |
|  | No | 68 | 35 | 33 |  |  |
| KPS | ≤80 | 68 | 32 | 36 | 0.599 | 0.562 |
|  | 90-100 | 44 | 24 | 20 |  |  |
| T stage | T1–2 | 36 | 18 | 18 | 0.000 | >0.999 |
|  | T3–4 | 76 | 38 | 38 |  |  |
| N stage | N0–2 | 51 | 26 | 25 | 0.036 | >0.999 |
|  | N3 | 61 | 30 | 31 |  |  |
| Metastasis status | MPE only | 36 | 18 | 18 | 0.000 | >0.999 |
|  | MPE+ Other metastasis | 76 | 38 | 38 |  |  |
| Other Metastases |  |  |  |  |  |  |
| Bone | Yes | 48 | 24 | 24 | 0.000 | >0.999 |
|  | No | 64 | 32 | 32 |  |  |
| Brain | Yes | 22 | 11 | 11 | 0.000 | >0.999 |
|  | No | 90 | 45 | 45 |  |  |
| Lung | Yes | 33 | 15 | 18 | 0.387 | 0.679 |
|  | No | 79 | 41 | 38 |  |  |
| Liver | Yes | 6 | 3 | 3 | - | >0.999 |
|  | No | 106 | 53 | 53 |  |  |
| Adrenal | Yes | 8 | 4 | 4 | - | >0.999 |
|  | No | 104 | 52 | 52 |  |  |
| Other | Yes | 19 | 9 | 10 | 0.063 | >0.999 |
|  | No | 93 | 47 | 46 |  |  |
| involving organ≤3 | Yes | 45 | 22 | 23 | 0.037 | >0.999 |
|  | No | 67 | 34 | 33 |  |  |
| Actionable mutation types | EGFR-M | 97 | 49 | 48 | 0.077 | >0.999 |
|  | ALK-P | 15 | 7 | 8 |  |  |
| Systematic chemotherapy | Yes | 47 | 27 | 20 | 1.796 | 0.251 |
|  | No | 65 | 29 | 36 |  |  |
| EGFR-TKIs | Gefitinib^1^ /Icotinib^2^ /Erlotinib^3^ | 80 | 40 | 40 | 0.049 | >0.999 |
|  | Osimertinib after 1/2/3 | 17 | 9 | 8 |  |  |
| ALK -TKIs | Crizotinib | 12 | 5 | 7 | - | 0.569 |
|  | Alectinib/ Alectinib after Crizotinib | 4 | 2 | 1 |  |  |
